# COVID-19 vaccination in psoriasis patients receiving systemic treatment: A prospective single-center study

**DOI:** 10.3389/fimmu.2023.1107438

**Published:** 2023-03-16

**Authors:** Georg Christian Lodde, Frederik Krefting, Jan-Malte Placke, Lea Schneider, Melanie Fiedler, Ulf Dittmer, Jürgen Christian Becker, Stefanie Hölsken, Dirk Schadendorf, Selma Ugurel, Wiebke Sondermann

**Affiliations:** ^1^Department of Dermatology, University Hospital Essen, University of Duisburg/Essen, Essen, Germany; ^2^Institute for Virology, University Hospital Essen, University of Duisburg/Essen, Essen, Germany; ^3^Translational Skin Cancer Research (tscr), University of Duisburg/Essen, Essen, Germany; ^4^German Consortium for Translational Cancer Research (DKTK), Partner Site Essen/Düsseldorf, Essen, Germany; ^5^Institute of Medical Psychology and Behavioral Immunobiology, University Hospital Essen, University of Duisburg/Essen, Essen, Germany

**Keywords:** COVID-19 vaccines, seroconversion, psoriasis, psoriasis treatment, biologics

## Abstract

**Background:**

The rate of seroconversion after COVID-19 vaccination in patients with moderate to severe psoriasis requiring systemic treatment is poorly understood.

**Objectives:**

The aim of this prospective single-center cohort study performed between May 2020 and October 2021 was to determine the rate of seroconversion after COVID-19 vaccination in patients under active systemic treatment for moderate to severe psoriasis.

**Methods:**

Inclusion criteria were systemic treatment for moderate to severe psoriasis, known COVID-19 vaccination status, and repetitive anti-SARS-CoV-2-S IgG serum quantification. The primary outcome was the rate of anti-SARS-CoV-2-S IgG seroconversion after complete COVID-19 vaccination.

**Results:**

77 patients with a median age of 55.9 years undergoing systemic treatment for moderate to severe psoriasis were included. The majority of patients received interleukin- (n=50, 64.9%) or tumor necrosis factor (TNF)-α inhibitors (n=16, 20.8%) as systemic treatment for psoriasis; nine patients (11.7%) were treated with methotrexate (MTX) monotherapy, and one patient each received dimethyl fumarate (1.3%), respectively apremilast (1.3%). All included patients completed COVID-19 vaccination with two doses over the course of the study. Serum testing revealed that 74 patients (96.1%) showed an anti-SARS-CoV-2-S IgG seroconversion. While all patients on IL-17A, -12 or -12/23 inhibitors (n=50) achieved seroconversion, three of 16 patients (18.8%) receiving MTX and/or a TNF-α inhibitor as main anti-psoriatic treatment did not. At follow-up, none of the patients had developed symptomatic COVID-19 or died from COVID-19.

**Conclusions:**

Anti-SARS-CoV-2-S IgG seroconversion rates following COVID-19 vaccination in psoriasis patients under systemic treatment were high. An impaired serological response, however, was observed in patients receiving MTX and/or TNF-α inhibitors, in particular infliximab.

## 1 Introduction

Psoriasis is a chronic inflammatory disease occurring worldwide which leads to typical erythematosquamous skin plaques and affects about 2-3% of the total Western population (1, 2). Already since the 1990s, there has been increasing scientific evidence that psoriasis is a systemic inflammatory disease associated with various comorbidities (3–5). Multiple epidemiological studies have shown an increased prevalence of cardiovascular risk factors, increased prevalence of arterial hypertension (6–11), and cardiovascular diseases like myocardial infarction (12–15) in psoriasis. In addition, a large body of evidence revealed that psoriasis is associated with obesity (7, 16–19), insulin resistance (20) and diabetes mellitus (6, 7, 11, 21, 22). Accordingly, various studies could show that psoriasis is closely related to metabolic syndrome (7, 16, 23–25). As a result of cardiovascular and cardiometabolic comorbidity, patients with severe psoriasis were shown to have a decreased life expectancy of up to 5 years (26, 27). Psoriasis is also frequently associated with psychological comorbidities. Altogether psoriasis has a massive negative impact on patients’ quality of life. In a US-based interview study, e.g. 98% of psoriasis patients reported that their emotional lives were impaired by their disease, 94% felt their social life was restricted and 68% perceived their careers to be hindered (28). Depending on the screening methodology, depressive symptoms are described to be present in up to 28-55% of psoriasis patients (6, 7). Social stigmatization due to easily visible skin manifestations is a strong predictor of depressive symptoms in psoriasis patients (29). However, there is emerging evidence that systemic inflammation may represent a pathophysiologic link between the diseases (30–32). For the above-mentioned reasons, patients with psoriasis, in particular with a severe form of the disease, have an urgent need for efficient therapies. Nowadays, systemic therapy options for moderate to severe psoriasis include conventional systemic agents such as dimethyl fumarate and methotrexate (MTX), the small molecule apremilast (phosphodiesterase-4 inhibitor), and various biologics (33). With the establishment of targeted cytokine inhibitors, the efficacy and tolerability of systemic therapies for the treatment of psoriasis patients has been massively increased (34). Unprecedented response rates of about 60% in terms of complete skin clearance are possible today with some of the newer biologics targeting interleukin (IL)-23 or IL-17 (35, 36). However, the first in group biologics licensed for psoriasis were tumor necrosis factor (TNF)-α inhibitors. In terms of safety profile, the incidence of severe adverse events in psoriasis patients receiving TNF-α inhibitors is low (37). Though, large cohort studies showed infliximab to be associated with an increased risk of serious infections (38). In addition, TNF-α inhibitors harbor a risk for reactivation of latent infections such as tuberculosis (39, 40). The rate of serious infections was shown to be higher especially for new users of infliximab and adalimumab (41). In contrast, therapy with anti-IL-12/23 antibodies and IL-17 inhibitors generally does not seem to increase the risk of serious infections (41).

Only very few reports in the literature so far have addressed the question to which extent immunomodulatory, respectively immunosuppressive therapies, such as those currently used to treat patients with psoriasis, influence the response to COVID-19 vaccines, which were shown to lead to serological responses in over 90% of healthy individuals (42, 43). For example, a recent study by Mahil et al. investigated psoriasis patients in the UK undergoing systemic therapy with MTX or targeted biological monotherapy. Functional humoral immunity to a single dose of BNT162b2 was shown to be impaired by MTX but not by targeted biologics, whereas cellular responses were unaffected (44, 45).

The aim of the present study was to determine the anti-SARS-CoV-2-S IgG seroconversion rate after COVID-19 vaccination in patients under active systemic treatment for moderate to severe psoriasis in order to expand the knowledge on this highly relevant topic.

## 2 Patients and methods

### 2.1 Study design and patient eligibility

**Figure 1:**
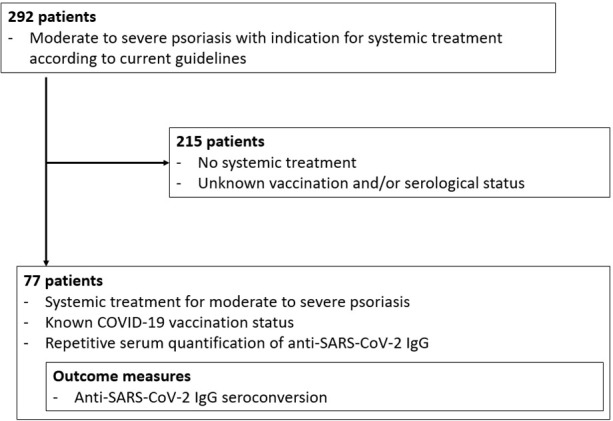
Patient flow chart.

This prospective single-center study of a consecutive sample of psoriasis patients under systemic treatment was performed from May 2020 until October 2021 at the Department of Dermatology, University Hospital Essen, Germany. Study outcome measures were anti-SARS-CoV-2-S IgG seroconversion and outcome of a potential COVID-19 disease. Data on patient characteristics, concomitant diseases, COVID-19 vaccination status, severity of psoriasis, systemic psoriasis therapy, and immunosuppressive comedication were collected. Leukocyte, neutrophil and lymphocyte counts were assessed at the time of first COVID-19 vaccination. Systemic treatment for psoriasis included biologics such as TNF-α inhibitors, IL-17A inhibitors, IL-23 inhibitors, the IL-12/23 inhibitor ustekinumab, the small molecule apremilast, as well as conventional therapies such as MTX and dimethyl fumarate. Concomitant diseases were evaluated using the modified Charlson Comorbidity Index (CCI) (46). Inclusion criteria were systemic treatment for moderate to severe psoriasis, completed COVID-19 vaccination corresponding to two sequential mRNA or viral vector vaccine applications, and repetitive anti-SARS-CoV-2-S IgG serum quantification (**Figure 1**). Anti-SARS-CoV-2-S IgG antibodies were measured at each time of consultation in our department.

### 2.2 Serum antibody testing

Measurement of IgG antibodies against SARS-CoV-2 spike protein (anti-SARS-CoV-2-S IgG) in patients’ sera was performed with the chemiluminescence assays SARS-CoV-2 S1/S2 IgG or SARS-CoV-2 TrimericS IgG, DiaSorin, Saluggia, Italy. The chemiluminescence analyzer LIASION-XL (DiaSorin) was used following the manufacturer’s instructions. The first assay is semi-quantitative as at the start of the study no standardized fully quantitative assays were available. The second assay is quantitative and adjusted to the upcoming WHO standard. Values >15 AU/ml corresponding to 39 BAU/ml, and values ≥33.8 BAU/ml were considered positive after first or second vaccination, respectively. Sensitivity/specificity for each assay are 94.4%/98.6% and 96.9%/100%, respectively. For this study, only qualitative results were used to document seroconversion.

### 2.3 Data analysis

Descriptive statistical analyses were performed using SPSSv26.0 (IBM, Armonk, NY, USA). The follow up time was defined as the period between first serum testing and last patient visit. The study was approved by the institutional ethics committee of the University Duisburg-Essen (21-10141-BO). It was conducted in accordance with the Declaration of Helsinki.

## 3 Results

### 3.1 Baseline characteristics

Within the studied period, 77 of 292 consecutive psoriasis patients presenting at the Department of Dermatology, University Hospital Essen, met the inclusion criteria (**Figure 1**). At a median age of 55.9 years (range 23.8-86.3 years), the majority of patients had a modified CCI of 0 (n=46, 59.7%, **Table 1**). Sixty-six patients (85.7%) were treated with biologics (TNF-α inhibitors (n=16), IL-17A inhibitors (n=21), IL-23 inhibitors (n=21), or an IL-12/23 inhibitor (n=8); nine patients received MTX (11.7%), one patient was treated with dimethyl fumarate (1.3%), and one patient received the phosphodiesterase-4 inhibitor apremilast. At the end of data collection in October 2021, after a median follow-up time of 12.7 months, none of the patients had developed a symptomatic COVID-19 disease or died from COVID-19. The mean time between the second vaccination and analysis of antibody levels was 3.9 weeks (range 0.4-23.0 weeks). None of the patients had been vaccinated more than 6 months before antibody analysis (mean time period between first vaccination and analysis: 12.2 weeks, range: 4.1-25.9 weeks).

**Table 1:** Patient characteristics.

	Total study cohort N (%)
**Total**	**77 (100.0)**
Median age, years (range)	55.9 (23.8-86.3)
Sex	
Female	39 (50.6)
Male	38 (49.4)
Comorbidities^1^	
Arterial hypertension	31 (40.3)
Obesity	15 (19.5)
Diabetes mellitus	10 (13.0)
Nicotine abuse	25 (32.5)
Charlson comorbidity index^2^	
0	46 (59.7)
1-2	23 (29.9)
≥3	8 (10.4)
Severity of psoriasis	
Moderate (Psoriasis Area and Severity Index <20)	66 (85.7)
Severe (Psoriasis Area and Severity Index ≥20)	11 (14.3)
Type of systemic treatment	
Biologics	66 (85.7)
TNF-α inhibitors	16 (20.8)
Adalimumab	9 (11.7)
Infliximab	3 (3.9)
Etanercept	2 (2.6)
Certolizumab	1 (1.3)
Golimumab	1 (1.3)
Interleukin inhibitors	50 (64.9)
Interleukin-12/23 inhibitor	8 (10.4)
Ustekinumab	8 (10.4)
Interleukin-17A inhibitors	21 (27.3)
Secukinumab	5 (6.5)
Ixekizumab	16 (20.8)
Interleukin-23 inhibitors	21 (27.5)
Tildrakizumab	9 (11.7)
Risankizumab	2 (2.6)
Guselkumab	10 (13.0)
Apremilast (Phosphodiesterase inhibitor)	1 (1.3)
Methotrexate	9 (11.7)
Dimethyl fumarate	1 (1.3)

### 3.2 Comparison of patients with and without seroconversion

After completed COVID-19 vaccination (corresponding to two sequential mRNA or viral vector vaccine applications), 74/77 patients (96.1%) achieved anti-SARS-CoV-2-S IgG seroconversion (**Table 2**). Seroconversion was reached in 64/66 patients treated with biologics. Three of these 64 patients received additional immunosuppressive comedication with MTX 5-10 mg per week in the context of psoriasis treatment (**Table 2**). COVID-19 vaccination led to seroconversion in 8/9 patients treated with MTX as main systemic psoriasis treatment, in 1/1 patient under therapy with dimethyl fumarate, and in 1/1 patient treated with apremilast (**Table 2**).

**Table 2:** COVID-19 vaccination and seroconversion.

	N (%)
Total	77 (100.0%)
Vaccination type (first and second vaccination)	
mRNA (2x mRNA-1273, n=4; 2x BNT162b24, n=52)	56 (72.7)
Viral vector (2x AZD1222)	9 (11.7)
Mixed (1x viral vector, 1x mRNA)	12 (15.6)
Immunosuppressive comedication for psoriasis	
None	72 (93.5)
Prednisolon 5mg/daily	1 (1.3)
Methotrexate	4 (5.2)
Methotrexate 5 mg/week	2 (2.6)
Methotrexate 7.5 mg/week	1 (1.3)
Methotrexate 10 mg/week	1 (1.3)
Anti-SARS-CoV-2-S IgG (serum)	
Positive prior to vaccination	0
Positive after vaccination (seroconversion)	74 (96.1%)
Biologics	64
TNF-α inhibitor	14
TNF-α inhibitor combined with methotrexate 5 mg/week	1
TNF-α inhibitor combined with methotrexate 7.5 mg/week	1
Interleukin inhibitors	50
Interleukin-12/23 inhibitor	8
Ustekinumab	8
Interleukin-17A inhibitor	21
Secukinumab	5
Secukinumab combinded with methotrexate 10 mg/week	1
Ixekizumab	16
Interleukin-23 inhibitor	21
Tildrakizumab	9
Risankizumab	2
Guselkumab	10
Apremilast (Phosphodiesterase inhibitor)	1
Methotrexate	8
Dimethyl fumarate	1
Not positive after vaccination (no seroconversion)	3 (3.9)
Biologics	2
TNF-α inhibitors	2
Infliximab	1
Infliximab combined with methotrexate 5mg/week	1
Methotrexate	1

In 3/77 completely vaccinated patients (3.9%) anti-SARS-CoV-2-S IgG antibodies could not be detected in sufficient amount in repeated serological tests after vaccination (anti-SARS-CoV-2-S IgG <33.8 BAU/ml) (**Table 2**).

The first patient without seroconversion, a 56-year-old female, was under therapy with the TNF-α inhibitor infliximab 5mg/kg Q7W i.v. and MTX 5 mg per week p.o. as an additional treatment. At the time of the first COVID-19 vaccination, she received her 46^th^ infliximab infusion. The interval between infliximab treatments was shortened from 8 to 7 weeks due to increasing arthralgia towards the end of the interval. The patient had no further relevant comorbidities (CCI 0). At the time of the first COVID-19 vaccination, laboratory parameters including lymphocytes, leukocytes and neutrophils were within the normal range. Repeated laboratory controls performed until the end of follow-up did not reveal any pathological parameters. After two vaccinations, the patient’s absolute anti-SARS CoV-2-S IgG was 25.7 BAU/ml.

The second patient failing seroconversion, a 57-year-old female, also received infliximab 5 mg/kg Q8W. In the past, the patient also had comedication with low-dose MTX, which was discontinued 9 months ago due to lymphopenia. At the time of the first COVID-19 vaccination, the patient received her 59^th^ infliximab infusion. The patient had no further relevant comorbidities (CCI 0). At the time of the first vaccination, the patient had decreased lymphocytes (0.90/nl). The lymphocyte counts remained decreased until the end of follow-up. After two vaccinations, the patient’s absolute anti-SARS CoV-2-S IgG was 26.5 BAU/ml.

The third patient failing seroconversion, an 86-year-old female, was treated with MTX 10 mg s.c. weekly. MTX treatment was initiated 4 weeks before the first COVID-19 vaccination. Lymphocytes, leukocytes and neutrophils were within normal ranges. The patient also suffered from diabetes mellitus type II, arterial hypertension and peripheral arterial occlusive disease of the lower legs. After two vaccinations, the patient’s absolute anti-SARS CoV-2-S IgG was 15.1 BAU/ml.

In the descriptive statistical comparison of the responder and non-responder group, no substantial differences were found. However, the median age of patients without seroconversion was slightly higher compared to patients with serological response (56.9 vs 55.6 years, **Table 3**). Of 13 patients receiving MTX as their main treatment, respectively comedication for psoriasis, 15.4% (n=2) failed to achieve seroconversion and of 16 patients receiving a TNF-α inhibitor as their main treatment for psoriasis 12.5% (n=2) did not reach seroconversion. The median values for leukocytes, neutrophils, and lymphocytes were similar in both groups at the time of the first COVID-19 vaccination (**Table 3**). However, patients who failed seroconversion (n=3) had lower median lymphocyte counts compared to patients who achieved seroconversion (1.6/nL (range 0.9-2.8) vs. 1.8/nL (range 0.6-3.8)).

**Table 3:** Characteristics of patients with and without anti-SARS-CoV-2-S IgG seroconversion.

	Seroconversion N (%)	No seroconversion N (%)
**Total**	**74 (100.0)**	**3 (100.0)**
Median age, years (range)	55.6 (23.8-86.3)	56.9 (55.3-84.7)
Sex		
Female	36 (48.6)	3 (100.0)
Male	38 (51.4)	0
Charlson comorbidity index		
0	44 (59.5)	2 (66.7)
1-2	22 (29.7)	1 (33.3)
≥3	8 (10.8)	0
Severity of psoriasis		
Moderate (Psoriasis Area and Severity Index <20)	63 (85.1)	3 (100.0)
Severe (Psoriasis Area and Severity Index ≥20)	11 (14.9)	0 (0.0)
Type of psoriasis treatment		
Biologics	64 (86.5)	2 (66.7)
TNF-α inhibitor	14 (18.9)	2 (66.7)
Interleukin inhibitor	50 (67.6)	0
Interleukin 12-/13 inhibitor	8 (10.8)	0
Ustekinumab	8 (10.8)	0
Interleukin 17A inhibitor	21 (28.4)	0
Secukinumab	5 (6.8)	0
Ixekizumab	16 (21.6)	0
Interleukin 23 inhibitor	21 (28.4)	0
Tildrakizumab	9 (12.2)	0
Risankizumab	2 (2.7)	0
Guselkumab	10 (13.5)	0
Methotrexate 10-15 mg/week	8 (10.8)	1 (33.3)
Apremilast (Phosphodiesterase inhibitor)	1 (1.4)	0
Dimethyl fumarate	1 (1.4)	0
Immunosuppressive comedication for psoriasis		
None	70 (94.6)	2 (66.7)
Prednisolon 5mg/daily	1 (1.4)	0
Methotrexate	3 (4.1)	1 (33.3)
Methotrexate 5 mg/week	1 (1.4)	1 (33.3)
Methorexate 7.5 mg/week	1 (1.4)	0
Methotrexate 10 mg/week	1 (1.4)	0
Methotrexate as main posriasis treatment or comedication		
Yes	11 (14.9)	2 (66.7)
No	63 (85.1)	1 (33.3)
Leukocytes (3.6-9.2/nL)		
Median (range)	7.3 (3.8-12.0)	7.0 (5.8-8.1)
Decreased		0
Within the normal range		3 (100.0)
Increased		0
Not available	6 (8.1)	0
Neutrophils (1.7-6.2/nL)		
Median (range)	4.4(2.2-8.5)	4.5 (2.4-6.3)
Decreased	0	0
Within the normal range	59 (79.7)	2 (66.7)
Increased	8 (10.8)	1 (33.3)
Not available	7 (9.5)	0
Lymphocytes (1.0-3.4/nL)		
Median (range)	1.8 (0.6-3.8)	1.6 (0.9-2.8)
Decreased	3 (4.1)	1 (33.3)
Within the normal range	58 (78.4)	2 (66.7)
Increased	6 (8.1)	0
Not available	7 (9.5)	0

## 4 Discussion

One of the main intentions of the presented work was to shed more light on the question whether the systemic agents currently used for the treatment of patients with moderate to severe psoriasis negatively affect the serological response to COVID-19 vaccines. Our results showed that 96.1% of patients responded to COVID-19 vaccination in the sense of a seroconversion. The rate of seroconversion was found to be slightly reduced in patients receiving MTX and/or TNF-α inhibitors compared to those under therapy with dimethyl fumarate, apremilast and biologics targeting IL-17 or IL-12/23.

Serological response rates to COVID-19 vaccines have been reported to exceed 90% in healthy individuals (42, 43), and neutralizing antibody levels were shown to be highly predictive of immunologic protection from symptomatic SARS-CoV-2 infection (47). Yet, neutralizing assays are complex and time-consuming. For reasons of practicability, we did not assess neutralizing antibody levels but IgG antibodies against the SARS-CoV-2 spike protein. However, data from the literature found a strong correlation between IgG and neutralizing antibodies at least within a 6-month period after the second dose of vaccination (48). In our cohort, none of the patients had been vaccinated more than 6 months before the analysis of antibodies. In addition to the measured high rates of seroconversion, the fact that no patient of our cohort reported an infection by SARS-CoV-2, COVID-19, or even died from COVID-19 confirmed that an effective protection was established by the vaccinations in nearly all cases. To the best of our knowledge, only very few comparable data were published. Mahil et al. (44, 49) for example measured the serological response of COVID-19 vaccines in 67 psoriasis patients on active systemic treatment. The authors reported that all patients showed seroconversion after completed vaccination consisting of two doses, which fits in well with our findings (44, 49). Other studies also presented similar findings (50–56). The main results of other studies that also investigated the serological response to COVID-19 vaccination in psoriasis patients are summarized in **Table 4**

**Table 4:** Results of other studies investigating the serological response to COVID-19 vaccination in psoriasis patients.

Reference	Total number of psoriasis patients and healthy controls finally investigated	Therapies	Antibody response in psoriasis patients under therapy with biologics N (%)	Antibody response in psoriasis patients under therapy with MTX as main or co-medication N (%)
Mahil et al., Lancet Rheumatol. (49)	64 patients and 15 controls	MTX n=14 TNF-α inhibitor n=18 IL-17 inhibitor n=13 IL-23 inhibitor n=19	50 (100.0) following the second dose of BNT162b2 vaccination	14 (100.0) following the second dose after BNT162b2 vaccination
Marovt et al., Clin Exp Dermatol. (50)	32 patients and 22 controls	TNF-α inhibitor n=7 IL-12/23 inhibitor n=11 IL-17 inhibitor n=6 IL-23 inhibitor n=8	32 (100.0) following the second dose of BNT162b2 vaccination	N/A
Graceffa et al., Front Med (Lausanne). (51)	45 patients and 45 controls	TNF-α inhibitor + MTX n=4 TNF-α inhibitor n=17 IL-12/23 inhibitor n=7 IL-17 inhibitor n= 5 IL-23 inhibitor n=12	44 (97.8)^1^ following the second dose of BNT162b2 vaccination	3 (75)^1^ following the second dose of BNT162b2 vaccination
Piros et al., Dermatol Ther. (52)	102 patients and 55 controls	TNF-α inhibitor n=57 IL-12/23 inhibitor n=28 IL-17 inhibitor n=16 IL-23 inhibitor n=1	102 (100.0) following the second dose of BNT162b2 or mRNA-1273 vaccination	N/A

1: One female patient on combination therapy (infliximab + methotrexate) did not respond to the double dose of vaccine.

IL, Interleukin; MTX, Methotrexate; N/A, Not applicable.

Two of three patients without seroconversion received MTX. MTX is a widely used immunosuppressant for the treatment of various immune-mediated inflammatory diseases. Its safety profile includes leucopenia/pancytopenia and proneness to infections (57). In previous studies, it has been shown that the response to COVID-19 vaccination can be negatively affected by MTX (58–60). In our cohort, patients who received MTX as their main systemic psoriasis treatment or as comedication showed an impaired seroconversion rate of only 84.6% after COVID-19 vaccination. Haberman et al. even found a strongly decreased anti-SARS-CoV-2 seroconversion rate of 62.2% in patients with immune-mediated inflammatory diseases receiving MTX (59). This negative effect of MTX on seroconversion rates is also known from other types of vaccines such as influenza, pneumococcal or tetanus (60–65).

Additionally, two of the three patients who did not achieve seroconversion after COVID-19 vaccination were under therapy with TNF-α inhibitors (n=1, infliximab monotherapy; n=1, infliximab plus MTX low-dose). As already stated above, TNF-α inhibitors, and especially infliximab, have an inferior safety profile in comparison with the newer cytokine inhibitors targeting IL-17 and IL-12/23, which is mainly due to a higher number of severe infections under TNF-α inhibitors (37, 38, 41) suggesting an impact on the immunologic surveillance of infectious diseases. The effect of TNF-α inhibitors on the immune system has already been reflected in decreased response rates observed in various studies on different vaccinations in the past (66–71). For example, reduced immune responses could be observed after influenza (68, 69), pneumococcal (70), and hepatitis B vaccinations (71) in patients receiving infliximab in comparison to therapy-naive patients.

It should be noted, that one of the serological non-responding patients was 86 years old, raising the question whether age may have an impact on COVID-19 vaccine response in psoriasis patients. Although our study only involved one older-aged patient with failed seroconversion, data from the literature indicate that older patients exhibit weaker or delayed immune responses to COVID-19 vaccines compared to younger patients (72). The aforementioned patient also had different concomitant diseases such as diabetes mellitus. Evidence regarding the efficacy of COVID-19 vaccines in patients with underlying diseases in general is still limited (73).

In our cohort, patients who failed seroconversion (n=3) had a lower median lymphocyte count compared to patients who achieved seroconversion. Avivi et al. reported statistically significantly lower seroconversion rates upon COVID-19 vaccination in patients with lymphopenia (74). However, it has to be considered that in our study the sample size of patients with failure of seroconversion was very low.

General limitations of our study include the relatively small number of patients, a high selection bias due to recruitment of patients from our highly specialized psoriasis center and the monocentric design. Furthermore, this study lacks a control group, neutralizing assays and cellular data such as B cell numbers as well as a quantification of T cell responses. Due to the highly dynamic development of new SARS-CoV-2 diagnostics, two test generations were used in this prospective longitudinal study. While the first assay used was semi-quantitative, the second assay was quantitative and adjusted to the upcoming WHO standard. As a result, it was not possible to present consistent quantitative data of antibody levels.

In the meantime, three vaccine doses against SARS-CoV-2 are widely considered as standard immunization and e.g. in Germany a fourth dose is even recommended for particular subgroups at risk (75). Thus, it would be of high interest to also investigate the seroconversion rates of patients with psoriasis under active systemic treatment systematically under these adapted conditions. There is already some data showing that initial non-responders can often achieve seroconversion in response to further vaccination, which is consistent with our own recent clinical experiences (76, 77).

For patients who do not mount antibody responses to COVID-19 despite repeated vaccination, a passive immunization against SARS-CoV-2 with neutralizing monoclonal antibodies is possible (78). Currently, the combinations of casirivimab/imdevimab (Ronapreve^®^) and tixagevimab/cilgavimab (Evusheld^®^) are approved by the European Medicines Agency for passive immunization against COVID-19 (79, 80). However, *in vitro* studies show that casirivimab/imdevimab does not result in relevant neutralization against the currently circulating Omicron variants (81–83). For this reason, the currently published German S1 guideline on SARS-CoV-2 pre-exposure prophylaxis recommends only the use of tixagevimab/cilgavimab for passive immunization, as in particular *in-vitro* studies of cilgavimab demonstrated the ability to neutralize omicron variants (78, 81–83).

## 5 Conclusion

The vast majority of psoriasis patients receiving systemic treatment achieved a seroconversion in response to two COVID-19 vaccinations. Seroconversion rate for patients under therapy with modern cytokine inhibitors targeting IL-17 or IL-23 was 100%. An impaired serological response was observed in patients who received MTX and/or TNF-α inhibitors, respectively. Larger real-world studies are needed to confirm our preliminary findings.

## Data availability statement

The original contributions presented in the study are included in the article/supplementary material. Further inquiries can be directed to the corresponding author.

## Ethics statement

The studies involving human participants were reviewed and approved by Ethics committee of the University Duisburg-Essen (21-10141-BO). Written informed consent for participation was not required for this study in accordance with the national legislation and the institutional requirements.

## Author contributions

Conceptualization: SU, WS, GL, FK. Methodology: SU, MF, WS, GL, FK. Formal analysis: MF, SU, WS, GL, FK. Resources: UD, DS, WS, SU, GL, FK. Data curation, GL, SU, WS, FK. Writing - original draft preparation: GL, SU, WS, FK. Writing and editing: GL, FK, MF, UD, J-MP, JB, DS, WS, SU. Visualization: SU, GL, WS, FK. Supervision: WS. Project administration: GL, FK, SU, WS. All authors contributed to the article and approved the submitted version.
